# Heredity1Read before the New Hampshire Veterinary Medical Association, February 4, 1896.

**Published:** 1896-05

**Authors:** W. T. Russell

**Affiliations:** Nashua, N. H.


### HEREDITY.[1]

BY W. T. RUSSELL, V.S.,
NASHUA, N. H.

(Continued from page 269.)

ONE might say of the true thief as of the poet, "He is born, not made." Everything goes to prove that the large mass of crime is hereditary, and that a man inherits evil passions and propensities as well as special features. Hence the hopelessness of reforming habitual criminals, as acknowledged by prison-philanthropists. Now who are the criminal classes? They are a distinct race of beings, inhabiting a distinct quarter of most of our large cities, living in intemperance and vice, closely intermarrying, and producing a criminal and degenerate stock. Habitual criminals are true moral imbeciles. Many are found to be descended from an epileptic or insane ancestry, and crime is an outlet to their insane tendencies. They would go mad

[1] Read before the New Hampshire Veterinary Medical Association, February 4, 1896.

if they were not criminal. Science does not regard man as an abstract being endowed with the ability of doing right or wrong, and we cannot consider crime in all cases as a simple affair of yielding to a vicious impulse or evil passion which ought to have been easily controlled. We must recognize a border-line between insanity and crime; near one boundary we meet something of madness, more of sin; near the other boundary we meet something of sin, but more of madness.

In regard to freedom of will in connection with heredity, if the majority of men were asked, "Is man a free-will agent?" their answer would be, emphatically, yes. But let us see, especially, in connection with heredity there is great doubt whether man is a free-will agent. Heredity is a fatalistic character. Heredity and freedom of will are in direct opposition to one another. By free-will we are ourselves, by heredity of others. Those in favor of free-will say, "I have an innate sense of my freedom of will;" but they ignore the fact that every mental state is determined by organic conditions, and that this very innate sense of freedom comes directly under the head of fatalism. It is a very interesting fact in this connection that statistics show that all acts commonly supposed to be the result of free-will, such as murders, thefts, marriages, and divorces, are about exactly the same year after year, allowing for the increase of population. In France, during the last twenty years, the number of criminals varied only from six to eight thousand. This reference amounts almost to a digression, but I have made it in hope of bearing out the argument that hereditary influence has scarcely any limit. Personally, I admit that often in thinking over this subject the idea will suggest itself, Are not we, ourselves, and the animals we have to deal with, simply passive agents in the carrying out of that great law of evolution in which heredity plays so prominent and subtle a part?

Now, gentlemen, having devoted too much attention, perhaps, to this portion of the subject, there remains to be treated the still more important application of it to diseased conditions. If the deductions to be drawn from the foregoing are correct, then how forcible must be the law of heredity in its application to the transmission of diseases? In this connection I take it for granted the interest of the meeting will centre itself chiefly upon the hereditary unsoundness in horses, particularly in relation to those affections upon which opinions differ, and it is to this part of the subject that I would direct your attention. For convenience I have grouped the conditions referred to into respiratory, nervous, bone, and blood affections, and, in addition, a few others that are not easily included in the above list.

We will commence with the respiratory division. Roaring and its variations, whether from atrophy of nerve or muscle, or from chronic thickening of the mucous membrane, have perhaps received as general a condemnation as any hereditary taint which may be thought of. On the other hand, broken wind is a much-disputed subject among the most eminent members of the profession. If we go to the human subject, and take its nearest analogy, asthma, you will find it evident that the medical profession is decided in its opinion on this point. Dr. Williams, in a paper on asthma, says: "Heredity can be traced in about 40 per cent. of the asthmatics, though the tendency may not show itself until late in life. The characteristic form of chest is often transmitted from parents to children, and even when this is not so a disposition toward asthmatic symptoms in catarrhal attacks is often seen in the children of asthmatics." I believe this quotation to be applicable to our equine patients in every line.

The nervous division does not admit of as much discussion, as for our purpose it includes epilepsy and stringhalt. Epilepsy, perhaps, would readily be admitted of a strongly hereditary tendency, but the fact of the impossibility of its detection in an ordinary examination for a time renders it beyond our pale. In regard to stringhalt, there is some difference of opinion, but grant the pathology of stringhalt as involving the nervous system, and I think it is unquestioned, and of all the hereditary affections those of the nervous system stand preeminent. Admit this, and a good case for the heredity of stringhalt is at once made out.

We next come to the bony enlargements about which opinions are so undivided. Spavin and splint have long been recognized as hereditary diseases; notoriously so, too, has sidebones, an affection well termed a curse among draft-horses in cities. The hereditary tendency of ring-bone I believe to be almost equally powerful, though there are disputes on this point. Navicular disease, which may, perhaps, be well included here, is, I think, on all sides now acknowledged to be hereditary. I have seen well-developed navicular disease in an unbroken colt.

Of the foregoing, splint is the only disease about which diversity of opinion exists as to the rejection of a sire for breeding purposes. Doubtless, in most cases, the risk involved is slight, but what I want to point out is this, the probable existence of a bony-enlargement diathesis, a condition in which the bones appear to be only waiting for a stimulus to throw out enlargements anywhere. Bony enlargements I think are, as a rule, pretty constant as to their production as to situation, yet one at times sees cases in which all the changes have been rung and inducing the idea that the exostoses develop just exactly in accordance with the amount of exertion devolving on the various limbs and positions. What I am leading up to is this, that the possessor of a large splint may probably, but not necessarily, be the possessor of such diathesis, and hence is an undesirable animal for breeding purposes. I raise the point simply as one allowing of consideration.

Of the bursal enlargements, throughpin has claimed the widest attention. Prof. Williams, with a very wide experience among Clydesdales, is very strong in his belief as to the heredity of this disease. Personally, I see no reason why the tendency to bursal inflammation and extra secretion is not equally transmissible with other diseases. From this standpoint, windgalls must of necessity be included in the list, though their frequency must be greatly augmented by conformation and here let me state my opinion, shortly, that distention of the capsule of the true hock-joint (bog-spavin) is equally transmissible.

Certain morbid conditions of the blood seem to be imparted to progeny by parents almost unfailingly. Grease is one of the most important, and its presence should warrant the rejection of its possessor. I think, also, eczema, although Erasmus Wilson says, "it is hereditary only in the sense of the transmission of natural tendencies from parent to offspring, and not by virtue of any special virus." Lymphangitis is open to discussion, although on this point I may mention that Faryer, in an article on "Elephantiasis in the Human Subject," says: "Richardson found that of 236 persons 73 per cent. had one or both parents affected." Mallenders and sallenders have been suggested as diseases which are hereditary. I would ask, Has it been positively decided that these diseases arise from any morbid condition of the skin or blood? Some believe that they are parasitic in their origin, and until this is settled it is hardly fair to ascribe it to heredity in any degree. Perhaps the members present may help me to a conclusion.

Among the diseases of the eyes we have, fortunately for our patients, not so wide a field for operation as the sister profession. By ourselves cataract is condemned as hereditary by a most unanimous voice; so, too, I think should be some forms of ophthalmia. As illustrative of the extreme delicacy of the law of heredity in its dealing with the human eye let me instance the frequency with which even a slight cast, not amounting to a squint, in the eye of a parent is reproduced throughout a long family.

The heredity of curb, though disputed, will probably hold good on examination, though at this time I am inclined to consider it due more to the transmission of the conformation of the hock than to inherited weakness of either ligament, tendon, or sheath.

This brings us to the all-important question of conformation, the heredity of which in its application to unsoundness, to my mind, does not receive the attention it deserves. Here we have, on the one hand, certain diseases upon the existence of which veterinary examiners are advised, and rightly too, to reject their possessor; on the other hand certain conformations, in themselves far more serious than some unsoundnesses proper, and with even a stronger hereditary tendency, and upon which we have not the slightest hold whatever, and why? Simply because their possessor is technically sound. Furthermore, the more I consider this matter the more do I become convinced that the word "sound" in its usual application to horses is a pitfall and snare. It draws a hard-and-fast line, allows of little or no room for individual opinion, and frequently lands a purchaser with an undesirable animal, because the said individual, having a perfect and blind confidence in the significance of the word, has committed himself to buy the animal passing sound. If possible, I would drop the word from our veterinary vocabulary. I doubt not but that most of us have heartily wished the word in Jericho, in many instances, in the interest of a client.

To return to conformation, "feet and hocks" are the points which chiefly lay themselves open to attack in this connection.

Take the case of a horse with big, flat feet and the usually low, weak heels. With such feet, as a rule, a long journey on a hard road, or a sharp attack of purging, is all that is necessary to produce acute laminitis. In this regard there is the shape of the feet, particularly after they have undergone the changes incident to the disease, do I consider laminitis hereditary. Then again is the vexed question of conformation of hock. Take, for instance, the so-called "sickle-shaped" hock, the transmissibility of which cannot be denied, although there are notable exceptions. I ask you, Is it not the rule that, given a fair amount of work, curb is the almost inevitable result? What I hold, and that most strongly, is that we should have the power equally of rejecting animals for such conformations as well as for the diseases already mentioned. Contracted feet and curby hocks are both, I believe, inheritable malformations, but neither, with no lameness present, seems of sufficient importance to warrant an animal's rejection.

An affection upon which I have not as yet seen any comment, as to heredity, is corns. If we admit brittle feet, I think, though not that the two necessarily go together, we must also admit corns. But my paper is already too long and I will omit any consideration of hereditary vices or congenital malformations, and will close, hoping for an earnest discussion and thanking you all for your kind attention.

